# The Thirty-Sixth Annual Catalogue of the Baltimore College of Dental Surgery

**Published:** 1875-05

**Authors:** 


### EDITORIAL. ETC.

*The Thirty-Sixth Annual Catalogue of the Baltimore College of Dental Surgery* has been issued the past month, and is embellished with a fine engraving of the New College Building situated on the S. E. Corner of Eutaw and Lexington Streets, opposite the largest market in the city, and in one of the best locations that could have been selected. This New Building is the largest, most elegant, and best arranged structure devoted to "Dental Instruction" in the world, and was completed for this purpose in February, 1875. The engraving, made from a photograph, and which can be seen in the advertisement in this Number of the Journal, shows it to be a handsome four story building with Mansard Roof, containing large Lecture, Infirmary, Laboratory and other Rooms, with windows extending from the high ceilings very near to the floors, and all arranged with special reference to their uses, and supplied with every convenience necessary for such an institution, and for the comfort of the students.

The THIRTY-SIXTH regular course of instruction in this College will commence on Thursday, October 14th, 1875, and continue until in the following March. The first two weeks of the Session are devoted to Infirmary Practice and Preliminary Clinics Lectures; the Regular Lectures commencing on the first day of November, 1875.

The Faculty take pleasure in announcing the continued and increasing prosperity of this Institution. Through its history of *thirty-six* years of continued existence, amid all the trials and difficulties of such an undertaking, its present proud position is established over the whole of the civilized world.

Between seven and eight hundred students have graduated from this College, and its diploma is held in such high esteem that large sums of money have from time to time been offered for it by dental practitioners residing in foreign countries, who desire the honor as a pecuniary benefit, without regard to qualification. It is scarcely necessary to say that the Diploma of the Baltimore College cannot be purchased.

The Faculty feel assured that the course of study and requirements, as well as the result of the examinations, will convince all honest minds, that not only a very marked improvement has been brought about by the officers of this College, in their endeavors to elevate the standard of dental education, within the past five years, but that this, the oldest Dental College in the world, still maintains its world wide reputation, and while it is far superior to many of recent growth, is second to none in this or any other country.

The Baltimore College has entered upon a new era in its history, and the Faculty are determined to graduate those only who are thoroughly competent.

The Alumni and all interested in Dental Education are very cordially invited to be present, not only at the Commencement Exercises, but during the final examination of the candidates for graduation, which takes place during the week preceding the Commencement.

The plan of instruction is designed to be in every respect thoroughly practical, using all available means to secure a complete course of instruction in the practice as well as in the theory of dentistry.

The absolute necessity for ACTUAL PRACTICE as an element of instruction, is not lost sight of in the effort to impart the knowledge necessary for the guidance of the dentist. In acknowledgment of the importance of this branch of tuition, the Faculty will continue to give special attention to the organization of the Practical Department, which affords more and better advantages than any private office

It will be of interest to dental students to have their attention called to the fact that the New Infirmary and Laboratory will be regularly open during the Spring, Summer and Fall, daily, and that this department of the College will be free to all Matriculants throughout the entire year. The Infirmary and Laboratory during the spring and summer, and until the Regular Session commences, is in charge of the Professor of Dental Mechanism and Metallurgy, whose daily attention and presence will be of inestimable benefit to students, and who will in person demonstrate on all subjects connected with actual practice. The benefits of a large Infirmary practice to the dental student cannot be well overestimated ; advantages are had which are entirely unobtainable in any private dental office. What students most feel their deficiency in, and most lack the opportunity of obtaining under a preceptor, the actual handling of instruments, and of witnessing and attempting for themselves the filling of teeth, are here enjoyed in full. There is always abundant material for practice, and both Infirmary and Laboratory are well equiped with instruments and apparatus. The large and interesting Museum of the College is also open to the constant inspection of students, and a study of the many specimens of morbid anatomy cannot fail to be of essential service.

An invitation is extended to all who wish to see the advantages of this system of instruction, to visit the Infirmary and compare it if they desire, with institutions of like character in other cities. The advantages of the spring, summer and part of the fall instruction in the Infirmary and Laboratory are offered free of charge to those who desire to avail themselves of it.

